# Hydrogen sulfide enhances salt tolerance in sorghum by activating the chloroplastic AsA–GSH cycle to sustain photosynthesis

**DOI:** 10.3389/fpls.2025.1664076

**Published:** 2025-10-21

**Authors:** Chang Liu, Sitong Liu, Xin Hu, Xiaolong Shi, Chunjuan Liu, Lu Sun, Yufei Zhou

**Affiliations:** ^1^ College of Agronomy, Shenyang Agricultural University, Shenyang, China; ^2^ College of Life Engineering, Shenyang Institute of Technology, Shenyang, China

**Keywords:** sorghum, salt stress, hydrogen sulfide, AsA-GSH cycle, photosynthesis

## Abstract

Soil salinization poses a severe threat to global food security by reducing crop productivity, particularly in semi-arid regions where sorghum (Sorghum bicolor L.) is a major cereal crop. Hydrogen sulfide (H₂S) has recently been recognized as a signaling molecule involved in plant stress tolerance. However, its role in regulating the chloroplastic ascorbate–glutathione (AsA–GSH) cycle and photosynthetic performance in sorghum under salt stress remains unclear. To investigate the potential regulatory role of exogenous H₂S, sorghum seedlings were subjected to salt stress with or without sodium hydrosulfide (NaHS, an H₂S donor). Physiological, biochemical, and chlorophyll fluorescence parameters were assessed to evaluate growth performance, antioxidant capacity, and photosynthetic responses. The concentrations of reduced and oxidized forms of ascorbate (AsA/DHA) and glutathione (GSH/GSSG), together with the activities of key enzymes in the AsA–GSH cycle, were determined. Salt stress significantly inhibited sorghum seedling growth, enhanced reactive oxygen species (ROS) accumulation, and disrupted redox homeostasis. Exogenous H₂S alleviated these effects by stimulating the AsA–GSH cycle in chloroplasts. H₂S treatment maintained higher levels of reduced AsA and GSH while promoting moderate accumulation of DHA and GSSG, accompanied by elevated activities of ascorbate peroxidase (APX), glutathione reductase (GR), dehydroascorbate reductase (DHAR), and monodehydroascorbate reductase (MDHAR). Moreover, H₂S improved photosynthetic performance by maintaining chlorophyll content and chloroplast ultrastructure, optimizing chlorophyll fluorescence parameters, and protecting photosystem II (PSII) from photoinhibition. Enhanced electron transfer from the PSII reaction center to plastoquinone further indicated an improved capacity for energy dissipation under salt stress. These findings demonstrate that exogenous H₂S confers salt tolerance in sorghum by activating the chloroplastic AsA–GSH redox cycle and preserving photosynthetic efficiency. The study highlights H₂S as a critical mediator of chloroplast redox regulation, providing an effective strategy for enhancing sorghum resilience to soil salinization and promoting sustainable agricultural production.

## 1 Introduction

Soil salinization, a consequence of irrigation practices, climate change, and natural soil processes, has become a major threat to global food security by severely impacting crop productivity (Mishra et al., 2023). Salinity stress restricts crop productivity by inducing osmotic stress and ion toxicity, which disrupt water uptake, ion homeostasis, photosynthesis, and ultimately reduce growth and yield (Parihar et al., 2015). Sorghum (*Sorghum bicolor* L.), an important cereal crop known for its tolerance to various environmental stresses, is predominantly cultivated in regions prone to salt-affected soils (Liu et al., 2023; Wei et al., 2024). Salinity hampers sorghum growth by disrupting essential physiological processes, including photosynthesis (Rajabi Dehnavi et al., 2022; Yang et al., 2020) and antioxidant metabolism (Chen et al., 2022; Guo et al., 2022), leading to reduced yields and potential crop failures. During crop adaptation to salinity stress, the identification and exploitation of salt-tolerance genes—such as the *TALE* (Liang et al., 2025), the *CASPL* (Xue et al., 2024a, 2024b), and the *RALF* (Xue et al., 2024c) and *TEF* (Liu et al., 2024)—constitute a pivotal strategy for breeding salt-tolerant cultivars. At same time, the exogenous application of plant growth regulators represents another critical approach for modulating sorghum salt tolerance.

The photosynthetic apparatus is a primary target in plants under salt stress, with the thylakoid membrane and associated electron transport components being particularly sensitive (Zhang et al., 2010). Within the chloroplast, the AsA-GSH cycle acts as a shield for photosynthesis against the oxidative harm triggered by salt stress (Tan et al., 2022). Beyond enhancing plant resilience across various stress scenarios (Zhu et al., 2022), this cycle also plays a key role in scavenging reactive oxygen species (ROS) within plant cells (Wu et al., 2019). However, under salt stress, the AsA-GSH cycle in plants is significantly impaired (Prajapati et al., 2023), adversely affecting crucial cellular activities such as stomatal movement (Khan et al., 2021). Notably, Hydrogen sulfide (H_2_S) can counteract salt stress by activating antioxidants within the AsA-GSH cycle and boosting the ROS scavenging ability within wheat plant cells (Kaya et al., 2023). However, the precise regulatory mechanisms by which H_2_S influences the photosynthetic process and the antioxidant metabolic pathways in sorghum seedlings remain to be elucidated.

Hydrogen sulfide (H_2_S), an emerging gasotransmitter with significant physiological functions in plants, has demonstrated potential in modulating plant responses to abiotic stresses (Xuan et al., 2020). Functioning as a signaling molecule, H_2_S participates in a variety of plant processes, including regulating gene expression, modifying proteins post-translationally, and preserving cellular redox balance (Alvi et al., 2023). Recent studies have highlighted that H_2_S could be crucial in enhancing plant resilience to adverse stress by regulating antioxidant defense mechanisms and safeguarding photosynthetic machinery. For instance, exogenous H_2_S application can improve cabbage photosynthesis under black rot stress by reducing chlorophyll degradation, enhancing gas exchange, and upregulating Calvin cycle enzyme activities and gene expressions related to photosynthesis (Wang et al., 2024). In addition, exogenous application of H_2_S, has been widely employed to enhance drought tolerance in plants. Such treatments promote the accumulation of polyamines, soluble sugars, and glycine betaine, while simultaneously stimulating antioxidant enzyme activities. These changes collectively mitigate drought-induced osmotic and oxidative stress, thereby improving the adaptive capacity of plants under adverse conditions (Thakur et al., 2021). Cui et al. (2025) indicated that H_2_S enhances plant cold tolerance by activating antioxidant defense mechanisms and facilitating the accumulation. In addition, exogenous iron and H_2_S collectively enhance seedling growth, maintain pigment composition, and bolster the antioxidative defense system in tomato seedlings under NaCl stress by increasing endogenous H_2_S content and L-cysteine desulfhydrase activity (Subba et al., 2023). Also, exogenous H_2_S has been demonstrated to mitigate salt stress in cucumber seedlings through multiple mechanisms, including boosting photosynthesis, maintaining the AsA-GSH cycle, protecting mineral ion intake, reducing the Na^+^/K^+^, and activating the SOS and MAPK signaling pathways ((Luo et al., 2023). Thus, there is a significant interest in elucidating the role of H_2_S in regulating the physiological functions in sorghum under salt stress conditions.

The primary objectives of this experiment were to: (1) investigate how H_2_S influences the growth of sorghum seedlings under salt stress conditions; (2) determine the impact of H_2_S on the photosynthetic machinery; (3) examine the influence of H_2_S on the AsA-GSH cycle in chloroplasts, which serves as a vital antioxidant defense system. By elucidating these mechanisms, our research seeks to provide essential insights for developing strategies to improve crop resilience against soil salinization, ultimately contributing to crop production and sustainable agriculture.

## 2 Materials and methods

### 2.1 Experimental location

The experiment was carried out within an artificial climate chamber (model AR-41L3 Flex, Percival, HK) situated in the Sorghum Physiology Laboratory at the Agronomy College, Shenyang Agricultural University. Specifically, the experimental chamber was set to maintain a consistent temperature of 28°C throughout both day and night, with a photoperiod set at 12 hours, an illumination intensity of 280 μmol m^-2^s^-1^, and a relative humidity at 84%.

### 2.2 Experimental materials and design

Sorghum inbred line, SX44B, was utilized as the experimental material in this study. Homogeneous sorghum seeds were meticulously chosen and sanitized using a 5% sodium hypochlorite solution for a duration of 10 minutes, after which they were placed in Petri dishes. Subsequently, the seeds were germinated in an incubator with a constant temperature value maintained at 25°C for a period of 3 days. After germination, seedlings that exhibited robust growth were transferred into hydroponic boxes, with 16 seedlings per box. The seedlings were initially cultured in distilled water for 3 days, before being transferred to a 1/2 Hoagland nutrient solution for an additional 3 days. Upon reaching the stage where second sorghum seedling leaf fully expanded, they were exposed to stress treatment with a 200 mmol/L NaCl salt solution (Yu et al., 2025). Sodium hydrosulfide (NaHS) at a concentration of 50 μmol/L was used as a hydrogen sulfide (H_2_S) donor, and hypotaurine at 0.1 mmol/L served as an H_2_S scavenger (based on previous results). Following salt stress, H_2_S or the H_2_S scavenger was sprayed once daily for the three consecutive days, with each application consisting of 8 mL. The experiment consisted of five treatments: (1) plants not exposed to salt stress received an equivalent volume of distilled water (CK), (2) plants not exposed to salt stress and treated with H_2_S (CK+H_2_S), (3) salt-stressed plants that were sprayed with distilled water (S), (4) salt-stressed plants that were sprayed with H_2_S (S+H_2_S), and (5) salt-stressed plants that were sprayed with both H_2_S and the H_2_S scavenger (S+H_2_S+HT). Throughout the experiment, the nutrient solution was refreshed every three days, and after a 7-day period of salt stress, uniform sorghum seedlings were selected for the measurement of various parameters (Tao et al., 2021).

### 2.3 Morphological measurements

Uniformly developed plant seedlings were selected from each treatment and cleaned with distilled water. The moisture on the surface of seedlings was carefully removed using filter paper. Subsequently, the shoot and root parts were dissected using scissors. The lengths of both the shoot and root sections were then precisely measured with a calibrated ruler. The fresh weights of these parts were accurately determined using an electronic balance accurate to 0.001 grams. Following this, each part was individually placed into a brown paper envelope and subjected to drying in an oven at 80°C until reaching a constant weight. Ultimately, the dry weights of the shoots and roots were then measured, with three replicates for each treatment (Yu et al., 2024).

### 2.4 Determination of MDA and reactive oxygen species content

The determination of O_2_^-^ content followed the method described by (Luo et al., 2023). Specifically, 0.5 g of the first fully expanded leaves from sorghum seedlings were collected in an ice bath and homogenized in 2 mL of extraction buffer. The mixture was then centrifuged at 8000×g for a duration of 10 minutes at 4°C, and the resulting supernatant was carefully retrieved. Next, Subsequently, 1 mL of the supernatant was combined with 0.5 mL of phosphate buffer (50 mmol/L, pH 7.8) and 0.1 mL of hydroxylamine hydrochloride chemical solution (10 mmol/L), and the reaction mixture was shaken and incubated at 25°C for 20 minutes. Subsequently, the mixture was treated with 1 mL of para-aminobenzenesulfonic acid solution (58 mmol·L^−1^) and 1 mL of α-naphthylamine solution (7 mmol·L^−1^). After adding these reagents, the mixture was thoroughly combined and subjected to oscillation at 30 °C for a period of 30 minutes. Ultimately, an equal volume of chloroform was introduced into the mixture, which was then subjected to centrifugation at 10,000×g for 3 minutes. The supernatant was carefully extracted, and its absorbance was assessed at a wavelength of 530 nm. The concentration of O_2_^-^ was subsequently quantified by referring to a pre-established standard calibration curve.

The H_2_O_2_ content was determined according to the procedure reported by Moloi and van der Westhuizen (2006). Specifically, 0.1 g of the first fully expanded leaves from sorghum seedlings were ground in 5 mL of cold acetone. The resulting homogenate was centrifuged at 4°C for 15 minutes, and the supernatant was carefully decanted. To this supernatant, 0.5 mL of titanium tetrachloride (TiCl_4_) reagent was added. During the mixing process, 3.5 mL of 25% ammonium hydroxide (NH_4_OH) was added dropwise. The mixture was then centrifuged again at 4°C for 5 minutes. The supernatant was discarded, and the precipitate was washed thoroughly with 5 mL of acetone until it turned colorless. Finally, the precipitate was melted in NH_2_SO_4_ solution (20 mL), and the absorbance was determined at 415 nm.

Nitroblue Tetrazolium (NBT) and Diaminobenzidine (DAB) Staining: Leaf segments (6–8 cm) from the first fully expanded leaves of sorghum seedlings were collected, with four replicates per treatment. Samples were incubated in NBT or DAB solution in the dark at room temperature for 6 h, followed by decolorization in 95% ethanol at 40 °C for 16 h. The decolorized leaves were rinsed, blotted dry, and photographed.

The malondialdehyde (MDA) content was assessed using the thiobarbituric acid (TBA) method, as described by Jin et al. (2020). Specifically, a 0.5 g sample of the first fully expanded leaves from sorghum seedlings was homogenized in trichloroacetic acid (5 mL) while being kept in an ice bath to ensure low-temperature conditions. After homogenization, the mixture was centrifuged at 8000×g for 15 minutes at 4°C to separate the components. Next, the resulting supernatant (2 mL) was combined with 5 mL TBA solution (0.67%) and incubated in boiling water for 30 minutes, after which it was cooled in an ice bath. The mixture was then subjected to centrifugation at 10000×g at 4°C for 15 minutes. Finally, the absorbance was determined at wavelengths of 532 nm, 600 nm, and 450 nm, respectively, using a 0.67% TBA solution as the reference.

The determination of MDA content is expressed through the following formula:


MDA (μmol/g FW)=6.45 × (A532−A600)−0.56×A450

where A450, A532, and A600 denote the optical density readings at 450 nm, 532 nm, and 600 nm, respectively.

### 2.5 Chloroplast extraction and preparation

The extraction and preparation of chloroplasts from leaf tissues were conducted in accordance with the protocol detailed by Bhattacharya et al. (2020). The first fully expanded leaves were selected (1 g), washed, and dried to remove petioles and major veins. The leaves were then homogenized in phosphate buffer (2 mL) and passed through a 100 μm mesh filter. The filtrate underwent centrifugation at 3000×g for 10 minutes on two separate occasions. Following each centrifugation, the supernatant was removed, and the resulting pellet was retained. Further centrifugation was conducted at 200×g and 1000×g for 2 minutes respectively, and the pellets were discarded while the supernatant was taken. Finally, the supernatant was subjected to centrifugation at 3000×g for 10 minutes, and the resulting pellet was collected as the chloroplast fraction. The chloroplasts were resuspended in 400 μL of chloroplast suspension solution for use in subsequent experiments.

### 2.6 Determination of antioxidant substances in the AsA-GSH cycle

A 300 μL aliquot of chloroplast suspension was mixed with 1.2 mL of 6% perchloric acid that had been pre-cooled. This mixture was then processed via centrifugation at 14000×g for 10 minutes at a temperature of 4°C. The concentrations of Ascorbic acid (AsA) and Dehydroascorbic acid (DHA) were assessed using the technique detailed by Wang et al. (2012). In parallel, to measure the levels of Glutathione (GSH) and Oxidized glutathione (GSSG), another 300 μL of chloroplast suspension was combined with 1.2 mL of 5% sulfosalicylic acid and subjected to centrifugation under identical conditions.

### 2.7 Determination of antioxidant substances enzyme activities in the AsA-GSH cycle

The assays for antioxidant enzyme activities within the AsA-GSH cycle were conducted following the protocols provided by the respective commercial kits. Specifically, the chloroplast suspension was carefully decanted into a fresh centrifuge tube. The kits utilized for measuring Ascorbate peroxidase (APX), Glutathione reductase (GR), Dehydroascorbate reductase (DHAR), and Monodehydroascorbate reductase (MDHAR) were procured from Suzhou Greats Biotech Co., Ltd., located in Suzhou, China.

### 2.8 Observation of chloroplast ultrastructure

Before collecting the samples, the leaves were thoroughly rinsed with distilled water, and any residual surface moisture was gently absorbed using filter paper. Next, the leaves were carefully excised into slender strips, approximately 1 mm × 3 mm in dimension, using a sharp blade (while avoiding the veins), and stored in glass vials filled with 2.5% pentanediol. To ensure complete submersion of the leaves in the glutaraldehyde solution, the vials were carefully depressurized using a syringe. Subsequently, they were kept at a refrigerated temperature of 4°C for the fixation process. After 2 days, the samples underwent three successive rinses with phosphate buffer (pH 7.8), fixed in osmium tetroxide (1%) for 2 hours, rinsed again three times with phosphate buffer, and then subjected to a dehydration process (50% and 70% ethanol, 80% and 90% acetone, each concentration for 15 minutes, and finally treated three times with 100% acetone, each time for 30 minutes). Subsequently, the samples were embedded in a blend composed of epoxy propane and SPON-812, followed by polymerization in a controlled environment chamber for a duration of 12 hours. The samples were meticulously sliced into 50 nm ultra-thin sections utilizing a Leica EM UC7 ultramicrotome (Wetzlar, Germany). These sections were subsequently mounted onto copper grids and subjected to staining with uranyl acetate and lead citrate solutions. Finally, the detailed structural analysis was conducted using a Zeiss LSM 500 transmission electron microscope (Zeiss, Germany).

### 2.9 Determination of chlorophyll synthesis precursors

The quantification of 5-Aminolevulinic acid (ALA) was conducted according to the method outlined by Wang et al. (2021). Specifically, 2 g of the first fully expanded leaves from sorghum seedlings were ground in 6 mL of ice-cold acetic acid buffer (pH 4.6) and then centrifuged at 5000×g for 15 minutes at 4°C. 4 mL of the resulting supernatant were combined with 100 μL of ethyl acetate and incubated at 100°C for 10 minutes. An equal volume of Ehrlich’s reagent, which consists of 2% p-dimethylaminobenzaldehyde, 6% perchloric acid, and 88% acetic acid, was added. After a 10-minute incubation, the absorbance was measured at 554 nm.

The measurement of porphobilinogen (PBG) content was adapted from the protocol described by Bogorad (1962) with slight adjustments. In detail, 0.3 g of the first fully expanded leaves from sorghum seedlings were carefully removed, finely minced, and transferred to a mortar. The leaf tissue was then homogenized with 2 mL of extraction buffer (0.6 mol/L Tris-HCl, 0.1 mol/L EDTA, pH 8.2) while kept in an ice bath until a homogeneous mixture was achieved. This homogenate was subsequently transferred to a centrifuge tube and centrifuged at 12000×g for 15 minutes. The supernatant was carefully collected, and an equal volume of Ehrlich’s reagent was added. After incubating in the dark for 15 minutes, the absorbance was recorded at 553 nm.

To determine the levels of protoporphyrin IX (Proto IX), Mg-protoporphyrin (Mg-Proto IX), and protochlorophyllide (Pchl), 0.3 g of the first fully expanded leaves from sorghum seedlings were chosen and homogenized in a mortar with 25 mL basic acetone (80%). Following the removal of impurities through filtration, the absorbance was determined at 575 nm, 590 nm, and 628 nm, respectively. The concentrations were then calculated using the equations provided by Wu et al. (2018).

### 2.10 Determination of photosynthetic pigments

Uniform plants from each treatment were selected, and the first fully expanded leaves were selected. The leaf surface was cleaned thoroughly, after which the leaves were cut into smaller sections. Subsequently, 0.1 g of fresh leaves were weighed for each replicate, with a total of three replicates for each treatment. Samples were positioned in glass containers and fully immersed in 10 mL ethanol (95%). The containers were stored in complete darkness for 48 hours to facilitate the complete extraction of chlorophyll. Subsequently, the analysis was performed using a UV-VIS spectrophotometer (Tokyo, Japan). The specific wavelengths corresponding to the maximum absorption peaks for chlorophyll a, chlorophyll b, and carotenoids in 95% ethanol were identified as 665 nm, 649 nm, and 470 nm, respectively.

The formulas used for calculations are as follows:


$$\begin{array}{c} \text{Concentration of chlorophyll a}\left(\text{mg}\cdot {\text{g}}^{-1}\text{FW}\right):\\ Ca=13.95A665-6.8A649 \end{array}$$

$$\begin{array}{c} \text{Concentration of chlorophyll b}\left(\text{mg}\cdot {\text{g}}^{-1}\text{FW}\right):\\ Cb=24.96A649-7.32A665\; \end{array}$$

$$\begin{array}{c} \text{Concentration of carotenoids}\left(\text{mg}\cdot {\text{g}}^{-1}\text{FW}\right):\\ Cc=\left(1000A470-2.05Ca-114.8Cb\right)/248 \end{array}$$

Where A665 and others indicate the optical density of chlorophyll solutions at 665 nm, 649 nm, and 470 nm, respectively.

### 2.11 Determination of photosynthetic parameters

The core photosynthetic metrics assessed included the net photosynthetic rate (Pn), stomatal conductance (Gs), transpiration rate (Tr), and intercellular CO_2_ concentration (Ci). These measurements were obtained from the first fully expanded leaf of sorghum seedlings, utilizing a Li-6400 photosynthesis system (LI-COR, USA). Each treatment was assessed with four replicates. The measurement conditions were standardized to a light intensity of 1000 μmol·m^-2^·s ^-1^, a CO_2_ concentration of 385 ± 5 μmol·mol^-1^, and a temperature of 28°C.

### 2.12 Assessment of chlorophyll fluorescence characteristics

For the assessment, the topmost fully expanded leaf was chosen, with four replicates for per treatment. Following a thorough rinse with distilled water and gentle drying of surface moisture using blotting paper, the leaves were acclimated in the dark at ambient temperature for 30 minutes. Leaf images were captured by a FlourCam FC800-O/2020 chlorophyll fluorometer (Brno, Czech Republic). The experimental setup for fluorescence measurements was configured in this manner: the initial fluorescence (F0) was captured under low actinic light intensity of 0.1 μmol·m-²·s-¹. This was succeeded by a saturation pulse light (10000 μmol·m-²·s-¹ for 0.7 seconds) to measure the maximum fluorescence (Fm). Following a 15-minute acclimation period under a light intensity of 800 μmol·m-²·s-¹, the value of Fm’ was determined. From these readings, several essential parameters were extracted, including F0, Fm, the quantum efficiency of PSII (Fv/Fm), and non-photochemical quenching (NPQ).

### 2.13 OJIP curve and PQ pool measurement

The topmost fully expanded leaf was employed to assess chlorophyll fluorescence parameters, including OJIP curve and PQ pool, utilizing a DUAL-PAM-100 dual-channel fluorometer (WALZ, Germany). The measurements were conducted after the leaves had been dark-adapted for 30 minutes.

### 2.14 Statistical analysis

Data were organized using Excel 2021, and graphs were created with Graph Pad Prism 8. The data obtained from a minimum of three replicates are presented as mean ± SD. Variance analysis was conducted using SPSS 26.0, and differences between treatments were tested for significance using Duncan’s method. Differences among treatments that are statistically significant at the p< 0.05 level are denoted by distinct lowercase letters.

## 3 Results

### 3.1 Effect of exogenous H_2_S on the morphology of sorghum seedlings

**Figure 1:**
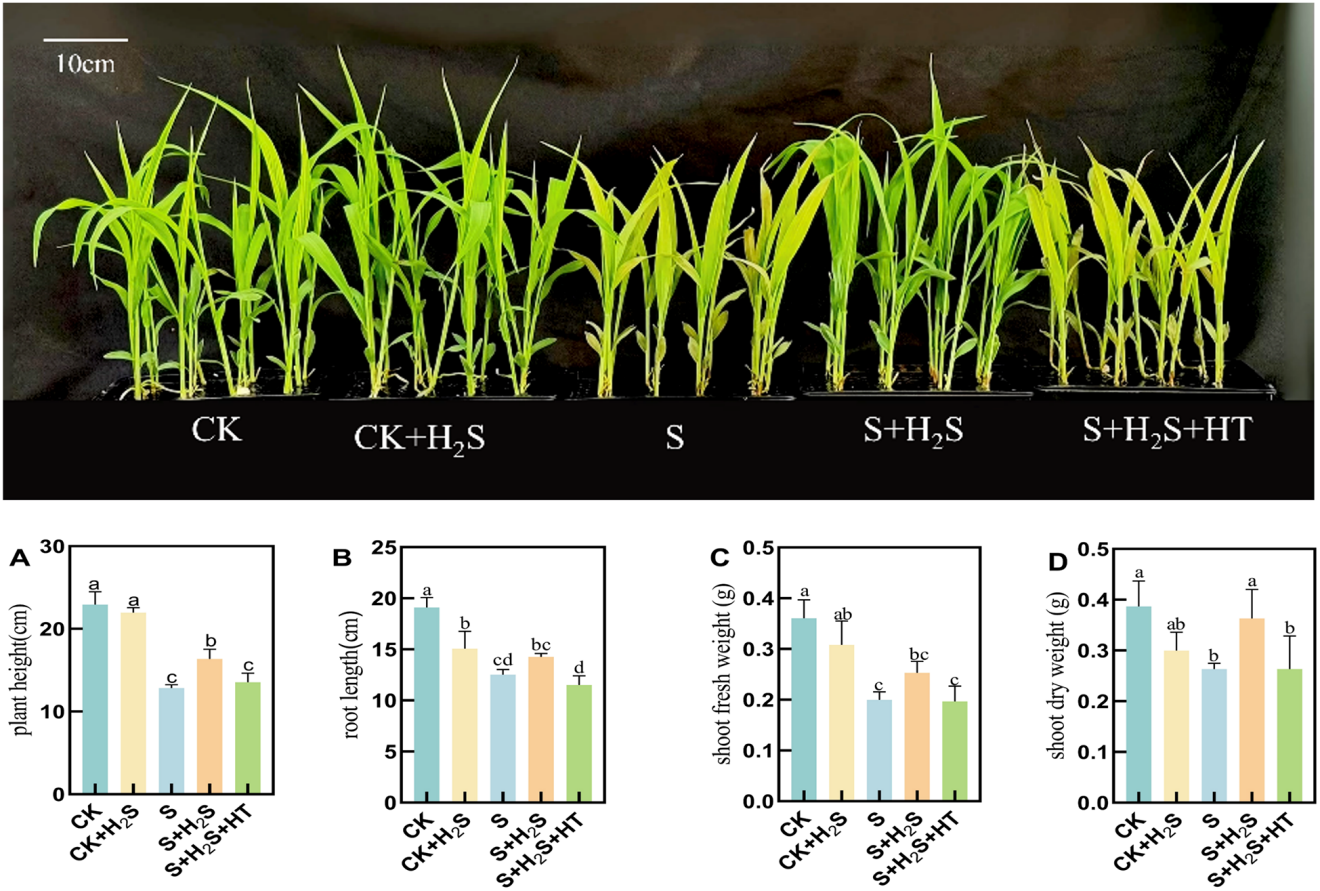
The phenotype of sorghum seedlings under salt stress, exogenous spraying H_2_S, H_2_S and HT, CK and CK+H_2_S. **(A)** Plant height, **(B)** Root length, **(C)** Fresh weight in shoots, and **(D)** Dry weight in shoots. Normal (CK); Normal spraying of H_2_S (CK+H_2_S); Salt stress (S); Salt stress spraying of H_2_S (S+H_2_S); Salt stress spraying of H_2_S and H_2_S scavengers (S+H_2_S+HT). The data represented the mean of the three replicates, and the different lower-case letters represented a significant difference at the 5% level (P<0.05).

Sorghum seedlings under salt stress exhibited a marked decline in growth (**Figure 1**). Specifically, compared to CK, salt-treated seedlings experienced substantial reductions in plant height (44.04%), root length (34.38%), shoot fresh weight (44.54%), and shoot dry weight (31.9%). In comparison with the S treatment, the S+H_2_S treatment increased the plant height, root length, shoot fresh weight, and shoot dry weight of the sorghum seedlings by 27.53%, 13.83%, 26.5%, and 37.97%, respectively. The S+H_2_S+HT treatment resulted in a decrease in plant height, root length, shoot fresh weigh, and shoot dry weight by 17.31%, 19.93%, 22.13%, and 27.52% compared to the S+H_2_S treatment. The aforementioned results indicated that foliar application of H_2_S significantly mitigated the adverse effects of salt stress on the growth of sorghum seedlings, thereby enhancing their overall development.

### 3.2 Effect of exogenous H_2_S on active oxygen species and MDA content of sorghum seedlings

**Figure 2:**
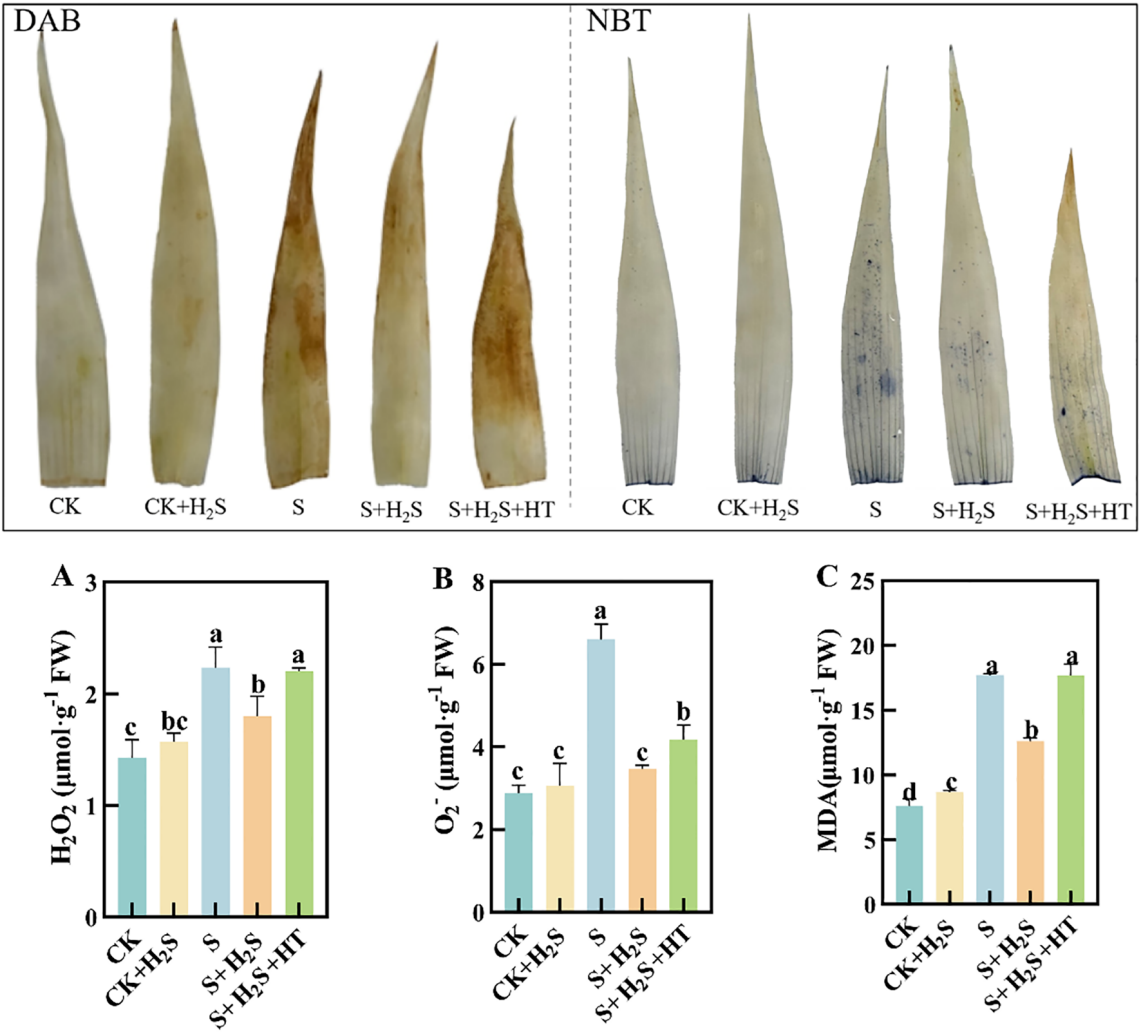
The DAB and NBT staining conditions and H_2_O_2_, O_2_^-^and MDA content under different treatments. **(A)** Hydrogen peroxide content, **(B)** Superoxide anion content, **(C)** Malondialdehyde content. Normal (CK); Normal spraying of H_2_S (CK+H_2_S); Salt stress (S; Salt stress spraying of H_2_S (S+H_2_S); Salt stress spraying of H_2_S and H_2_S scavengers (S+H_2_S+HT). The data represented the mean of the three replicates, and the different lower-case letters represented a significant difference at the 5% level (P<0.05).

To directly visualize the production of ROS in sorghum seedling leaves caused by salt stress, this study employed DAB and NBT staining solutions to specifically stain tissues containing H_2_O_2_ and O_2_^-^ in the sorghum seedling leaves. The intensity of leaf color correlates with the concentration of H_2_O_2_ and O_2_-, where a deeper color indicates higher levels of these reactive oxygen species. Compared to the CK, the S treatment significantly increased the content of O_2_^-^, H_2_O_2_, and MDA in the sorghum seedling leaves by 129%, 56.56%, and 132.61%, respectively (**Figure 2**). In contrast, the S+H_2_S treatment significantly reduced the content of O_2_^-^, H_2_O_2_, and MDA by 47.47%, 19.47%, and 28.88%, respectively, compared to the S treatment. Furthermore, the S+H_2_S+HT treatment resulted in notable increases in the content of O_2_^-^, H_2_O_2_, and MDA by 20.54%, 22.54%, and 40.29%, respectively, compared to the S+H_2_S treatment.

### 3.3 Effect of exogenous H_2_S on the contents of AsA, DHA, GSH and GSSG in chloroplasts of sorghum seedlings

**Figure 3:**
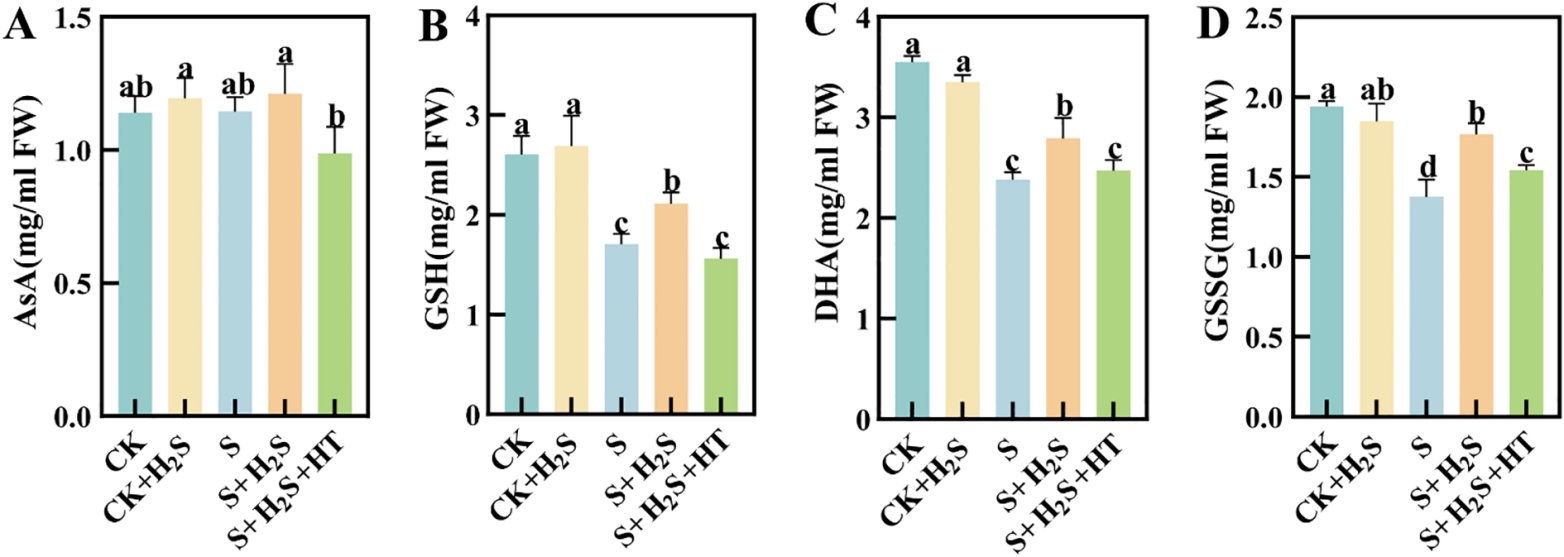
Effects of H_2_S spraying on chloroplast AsA, DHA, GSH, and GSSG contents of sorghum seedlings under salt stress **(A)** AsA content, **(B)** GSSG content, **(C)** DHA content, **(D)** GSSG content. Normal (CK): Normal spraying of H_2_S (CK+H_2_S): Salt stress (S): Salt stress spraying of H_2_S (S+H_2_S): Salt stress spraying of H_2_S and H_2_S scavengers (S+H_2_S+HT). The data represented the mean of the three replicates, and the different lower-case letters represented a significant difference at the 5% level (P<0.05).

Salt stress significantly impaired the functioning of the AsA-GSH cycle within the chloroplasts of sorghum leaves at seedling stage. When compared to CK, the S treatment led to a significant reduction in the levels of AsA and GSH within the chloroplasts of sorghum seedling leaves, with decreases of 0.49% and 34.43% (P<0.05), respectively (**Figures 3A, B**), while the contents of DHA and GSSG significantly decreased by 32.96% and 29.47%, respectively (**Figures 3C, D**). The S+H_2_S treatment increased these contents by 5.72%, 23.77%, 17.22%, and 28.46%, respectively, compared to the S treatment. In contrast, the S+H_2_S+HT treatment resulted in substantial declines in the levels of AsA, GSH, DHA, and GSSG by 18.47%, 26.15%, 11.36%, and 12.5%, respectively, when compared to the S+H_2_S treatment.

### 3.4 Exogenous H_2_S effectively increased APX, GR, DHAR and MDHAR activities in chloroplasts of sorghum seedlings

**Figure 4:**
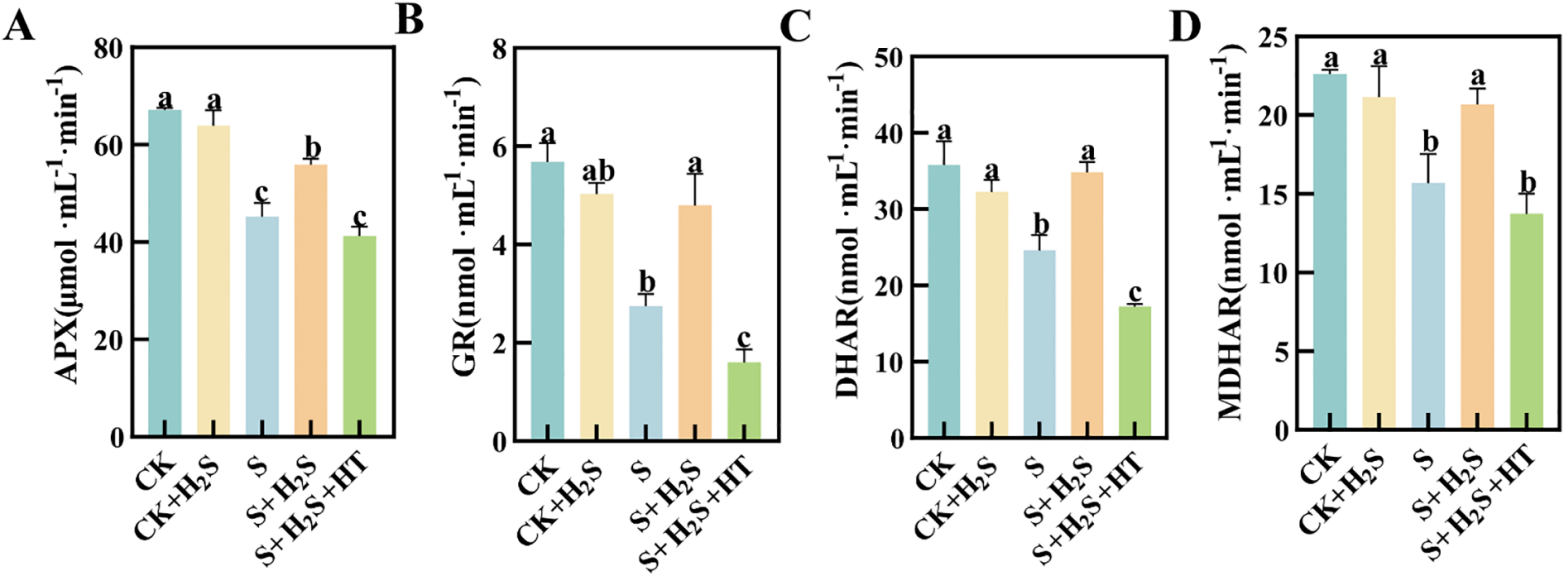
Effects of H_2_S spraying on chloroplast APX, GR, DHAR and MDHAR activities of sorghum seedlings under salt stress **(A)** APX activity, **(B)** GR activity, **(C)** MDHAR activity, **(D)** DHAR activity. Normal (CK): Normal spraying of H_2_S (CK+H_2_S): Salt stress (S): Salt stress spraying of H_2_S (S+H_2_S): Salt stress spraying of H_2_S and H_2_S scavengers (S+H_2_S+HT). The data represented the mean of the three replicates, and the different lower-case letters represented a significant difference at the 5% level (P<0.05).

Salt stress notably suppressed the activity of enzymes involved in the AsA-GSH cycle (**Figure 4**). Compared to CK, the activities of APX, GR, DHAR, and MDHAR in the chloroplasts of sorghum seedling leaves under S treatment significantly decreased by 32.72%, 51.6%, 31.28%, and 30.65%, respectively. In contrast, the S+H_2_S treatment resulted in substantial enhancements in the activities of APX, GR, DHAR, and MDHAR, with respective increases of 23.65%, 74.4%, 41.64%, and 31.88% compared to the S treatment. Furthermore, the S+H_2_S+HT treatment significantly reduced the activities of these enzymes by 26.19%, 66.59%, 50.51%, and 33.58%, respectively, when compared to the S+H_2_S treatment.

### 3.5 Effect of exogenous H_2_S on chloroplast structure of sorghum seedlings

**Figure 5:**
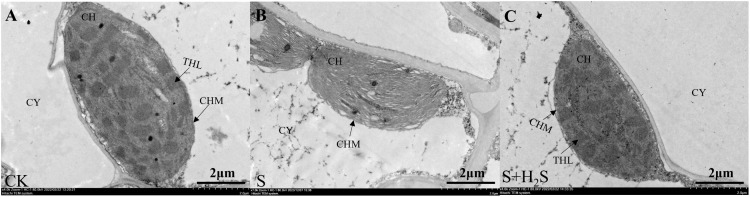
Effects of H_2_S spraying on the ultrastructure of chloroplasts in sorghum seedlings under salt stress. (CY) cytoplasm, (CH) chloroplast, (CHM) chloroplast membrane, (THL) thylakoid lamella. **(A–C)** indicate normal (CK), Salt stress (S) and Effect of salt stress spray H_2_S (S+H_2_S) on chloroplast ultrastructure (A-D×7000 magnification, scale =2 μm), respectively.

Transmission electron microscopy showed that chloroplasts in CK-treated seedling-age sorghum leaves were spindle-shaped, closely appressed against the cell walls, with intact double membrane structures and neatly stacked thylakoid grana that were clearly defined (**Figure 5**). In contrast, the chloroplasts under S treatment exhibited significant morphological alterations, with damaged chloroplast membranes and disorganized thylakoid grana, some of which were disintegrated and unclear. Compared to the S treatment, the S+H_2_S treatment maintained the integrity of the double membrane structure, and the thylakoid grana were more orderly arranged. This suggested that the foliar application of H_2_S can alleviate the damage to the chloroplast structure in sorghum seedling leaves caused by salt stress.

### 3.6 Effect of exogenous H_2_S on chlorophyll synthesis precursor contents of sorghum seedlings

**Figure 6:**
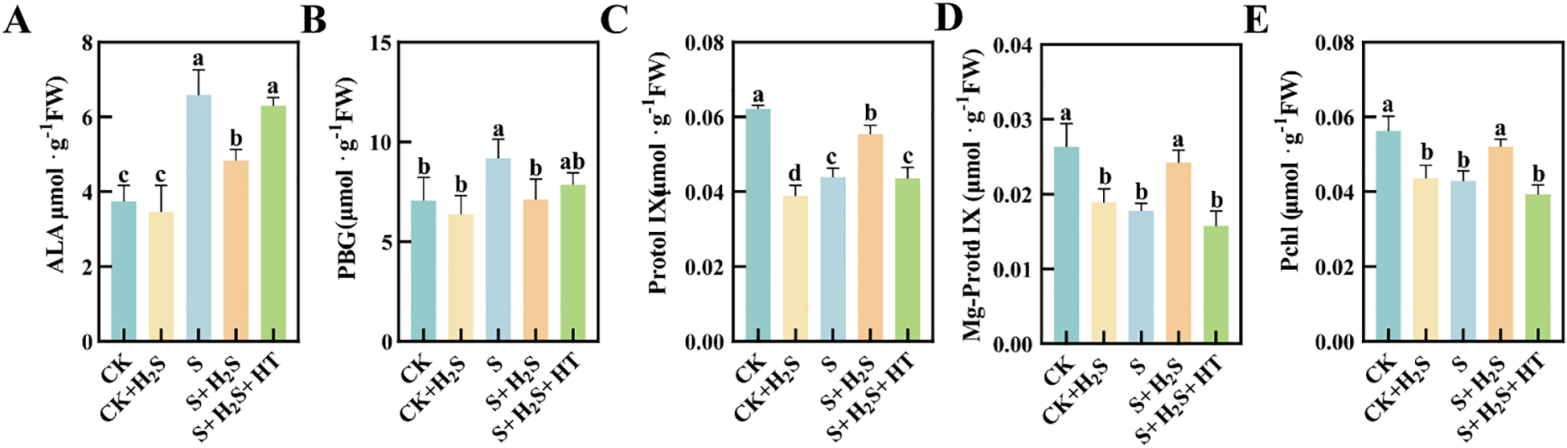
Effects of H_2_S spray on chlorophyll synthesis precursors of sorghum seedlings under salt stress **(A)** ALA, **(B)** PBG, **(C)** Protol IX, **(D)** Mg-Protol IX, **(E)** Pchl. Normal (CK): Normal spray H_2_S (CK+H_2_S); Salt stress (S); Salt stress spray H_2_S (S+H_2_S); Salt stress spray H_2_S and H_2_S scavenger (S+H_2_S+HT). The data represented the mean of the three replicates, and the different lower case letters represented a significant difference at the 5% level (P<0.05).

Salt stress led to elevated levels of ALA and PBG in sorghum seedling leaves (**Figures 6A, B**), while simultaneously reducing the contents of Protol IX, Mg-Protol IX, and Pchlide (**Figures 6C–E**). Compared to CK, the S treatment resulted in substantial increases in content of ALA and PBG by 76.06% and 29.96%, respectively. Conversely, the contents of Protol IX, Mg-Protol IX, and Pchlide significantly reduced by 29.3%, 32.39% and 23.78%, respectively. In comparison with the S treatment, the S+H_2_S treatment significantly reduced the content of ALA and PBG in sorghum leaves at the seedling stage by 26.61% and 22.53%, respectively, and increased the content of Protol IX, Mg-Protol IX, and Pchlide by 26.2%, 35.91%, and 21.3%, respectively. Furthermore, compared to the S+H_2_S treatment, the S+H_2_S+HT treatment increased the content of ALA and PBG by 30.35% and 10.33%, respectively, and significantly decreased the content of Protol IX (21.39%), Mg-Protol IX (34.89%), and Pchlide (24.51%).

### 3.7 Effect of exogenous H_2_S on chloroplast Chla, Chlb and Car contents of sorghum seedlings

**Figure 7:**
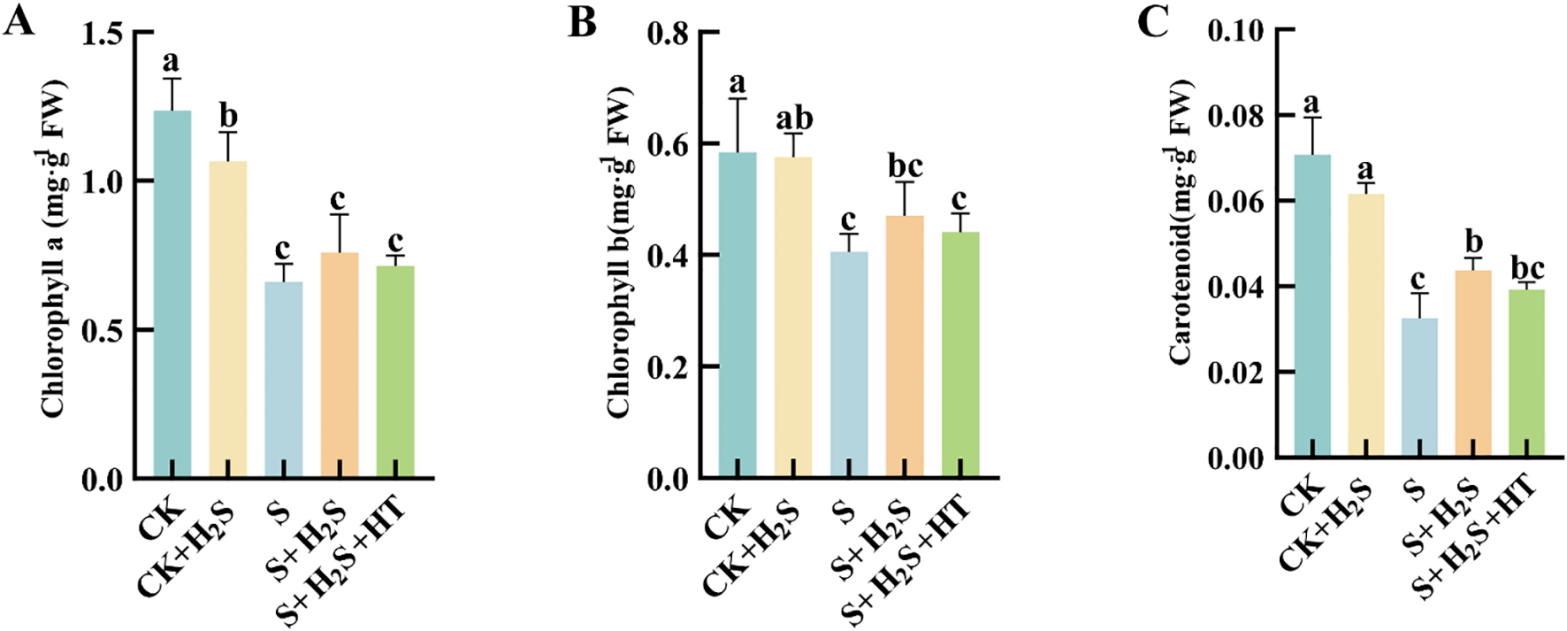
Effects of H_2_S spraying on Chla, Chlb and Car of sorghum seedlings under salt stress **(A)** Chla content, **(B)** Chlb content, **(C)** Car content. Normal (CK); Normal spraying of H_2_S (CK+H_2_S); Salt stress (S); Salt stress spraying of H_2_S (S+H_2_S); Salt stress spraying of H_2_S and H_2_S scavengers (S+H_2_S+HT). The data represented the mean of the three replicates, and the different lower-case letters represented a significant difference at the 5% level (P<0.05).

Under salt stress conditions, the concentrations of Chla, Chlb, and Car in the leaves of sorghum seedlings were substantially reduced (**Figure 7**). Compared to CK, S treatment led to substantial reductions in the contents of Chla, Chlb, and Car by 46.56%, 30.64%, and 53.99%, respectively. In contrast, compared to the S treatment, the S+H_2_S treatment resulted in notable increases in Chla (14.97%), Chlb (15.95%), and Car (34.21%). Furthermore, compared to the S+H_2_S treatment, the S+H_2_S+HT treatment caused a decrease in the contents of Chla (5.94%), Chlb (6.17%), and Car (10.19%).

### 3.8 Effect of exogenous H_2_S on chlorophyll fluorescence parameters of sorghum seedlings

**Figure 8:**
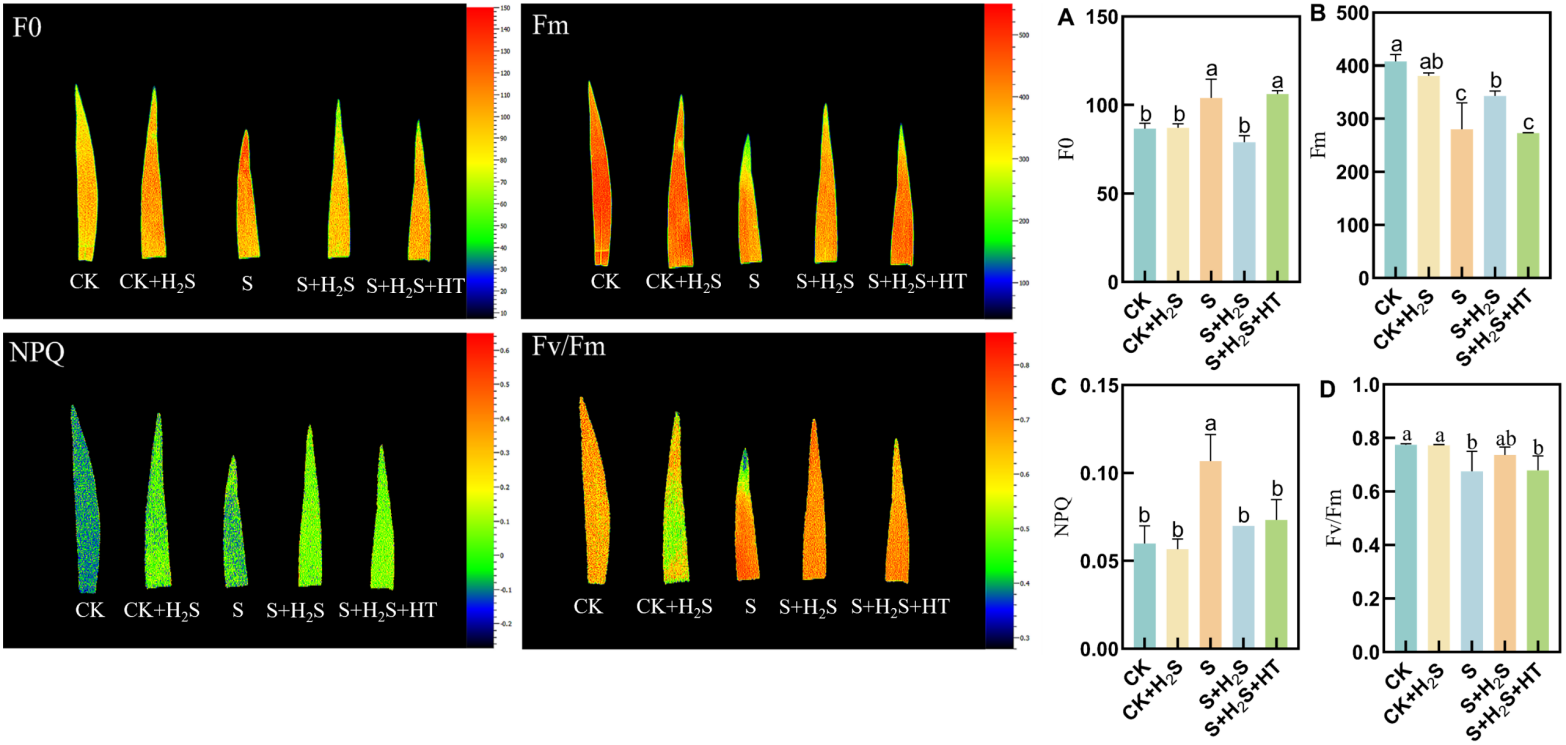
Effects of H_2_S spraying on F0, Fm, Fv/Fm of sorghum seedlings under salt stress **(A)** F0, **(B)** Fm, **(C)** NPQ, **(D)** Fv/Fm. Normal (CK); Normal spraying of H_2_S (CK+H_2_S); Salt stress (S); Salt stress spraying of H_2_S (S+H_2_S); Salt stress spraying of H_2_S and H_2_S scavengers (S+H_2_S+HT). The data represented the mean of the three replicates, and the different lower-case letters represented a significant difference at the 5% level (P<0.05).

The chlorophyll fluorescence parameters Fm and Fv/Fm in sorghum seedling leaves decreased, while F0 and NPQ increased when subjected to salt stress (**Figure 8**). Compared to CK, the Fm and Fv/Fm in the S treatment significantly decreased by 31.34% and 12.77%, respectively, while F0 and NPQ significantly increased by 20.01% and 76.66%, respectively. In comparison with the S treatment, the S+H_2_S treatment reduced F0 and NPQ by 24.02% and 33.96%, respectively, and increased Fm and Fv/Fm by 22.47% and 9.1%, respectively. Furthermore, compared to the S+H_2_S treatment, the S+H_2_S+HT treatment led to a decrease in Fm and Fv/Fm by 20.44% and 7.95%, respectively, and an increase in F0 and NPQ by 34.15% and 4.71%, respectively.

### 3.9 Effect of exogenous H_2_S on gas exchange parameters of sorghum seedlings

**Figure 9:**
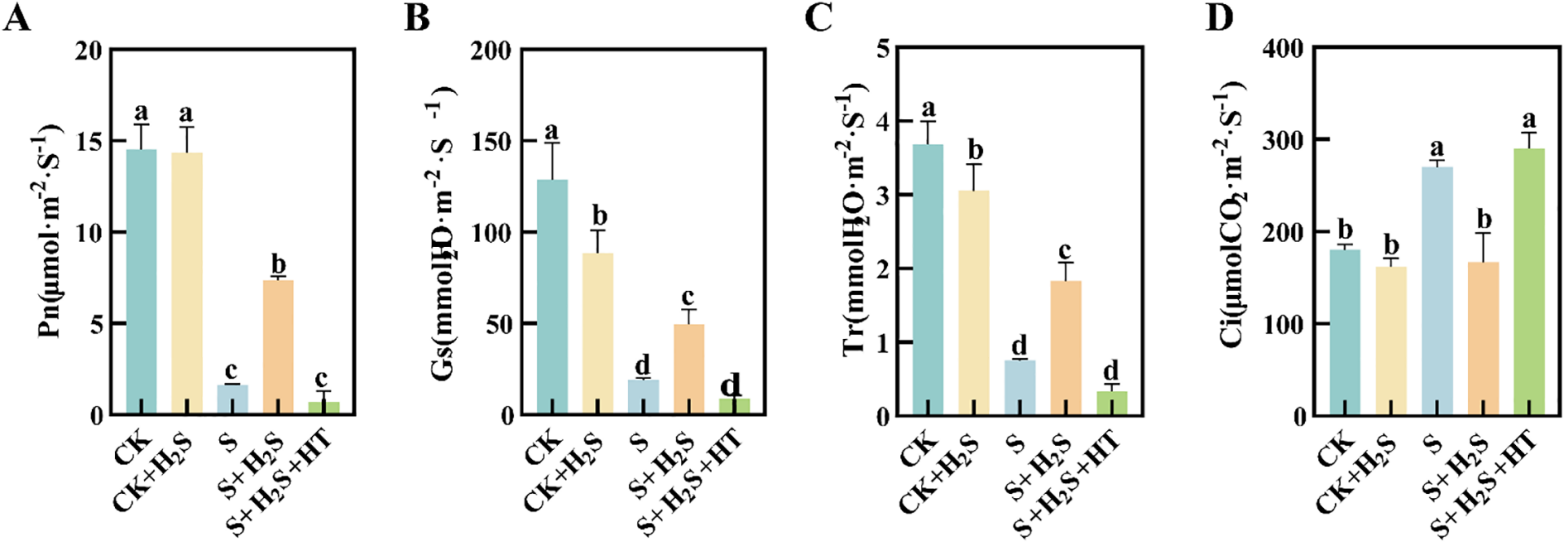
Effects of H_2_S spraying on Pn, Tr, Gs and Ci of sorghum seedlings under salt stress **(A)** Pn, **(B)** Tr, **(C)** Gs, **(D)** Ci. Normal (CK): Normal spraying of H_2_S (CK+H_2_S): Salt stress (S): Salt stress spraying of H_2_S (S+H_2_S): Salt stress spraying of H_2_S and H_2_S scavengers (S+H_2_S+HT). The data represented the mean of the three replicates, and the different lower-case letters represented a significant difference at the 5% level (P<0.05).

Sorghum seedling leaves showed reduced Pn, Gs, and Tr (**Figures 9A–C**) under salt stress, while Ci increased (**Figure 9D**). S treatment compared to CK significantly reduced Pn, Gs and Tr in sorghum seedling leaves by 88.76%, 79.63%, and 85.23%, respectively, and Ci increased significantly by 49.72%. In contrast, the S+H_2_S treatment significantly increased Pn, Gs and Tr by 351.02%, 144%, and 159.64%, separately, and significantly decreased Ci by 38.27% compared to the S treatment. Furthermore, compared to the S+H_2_S treatment, the S+H_2_S+HT treatment significantly reduced Pn, Gs and Tr by 90.49%, 81.78%, and 82.43%, respectively, and significantly increased Ci by 73.79%.

### 3.10 Impact of exogenous H_2_S on OJIP curve and PQ pool in sorghum seedlings

**Figure 10:**
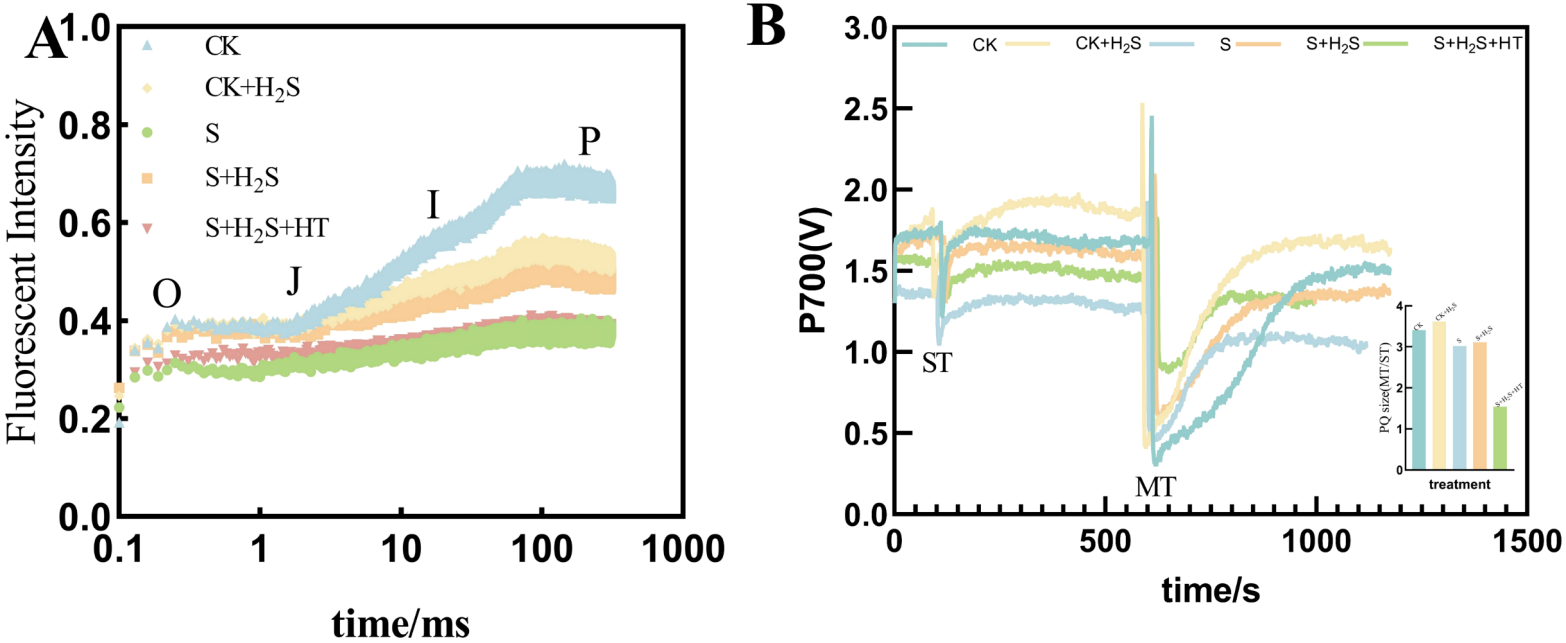
Effects of H_2_S spraying on OJIP curves and PQ pools of rapid chlorophyll fluorescence-induced kinetics of sorghum seedlings under salt stress **(A)** PQ library and **(B)** OJIP curves. Note: A single turnover saturation pulse analysis is performed to determine the P700 signal (ST), followed by a double turnover saturation pulse analysis (MT), the PQ library is oxidized, then the PQ size is MT area over ST area. Normal (CK); Normal spraying of H_2_S (CK+H_2_S); Salt stress (S); Salt stress spraying of H_2_S (S+H_2_S); Salt stress spraying of H_2_S and H_2_S scavengers (S+H_2_S+HT). The data represented the mean of the three replicates, and the different lower-case letters represented a significant difference at the 5% level (P<0.05).

Under salt stress, the shape of the OJIP curve of sorghum seedling leaves varied with the different treatments (**Figure 10A**). Compared to CK, the fluorescence signal intensity was significantly reduced in the S treatment. The S+H_2_S treatment increased Compared with S treatment, the fluorescence signal intensity of sorghum leaves at seedling stage increased significantly. The fluorescence signal intensity of leaves treated with S+H_2_S+HT showed no significant difference compared to the S treatment. However, the fluorescence signal intensity of leaves treated with S+H_2_S was higher than that of the S+H_2_S+HT treatment, particularly during the I-P phase, indicating a notable increase. Under salt stress, calculation of the MT and ST area ratio indicated a decrease in the PQ pool of sorghum seedling leaves (**Figure 10B**). Specifically, the size of the PQ pool was significantly diminished by 11.32% in the S treatment compared to the CK. Conversely, compared to the S treatment, the PQ pool size expanded by 3.01% in the S+H_2_S treatment.

## 4 Discussion

Salt stress severely impeded the growth of sorghum seedlings, as manifested by the reduction in plant height, root length, and biomass accumulation compared with control plants. Such growth inhibition is a common outcome of osmotic and ionic stress, which limits cell expansion and nutrient acquisition. Similar inhibitory effects of salinity on plant growth have been widely reported (Van Zelm et al., 2020). However, exogenous H_2_S application effectively alleviated these negative effects, highlighting its role as a growth-promoting factor under saline conditions. Conversely, the suppression of endogenous H_2_S abolished these benefits, further confirming its protective role in sorghum growth under salt stress (**Figure 1**).

Photosynthesis is particularly vulnerable to salinity, and our findings demonstrate that salt stress led to a pronounced decline in photosynthetic parameters, including net photosynthetic rate (Pn), stomatal conductance (Gs), and transpiration rate (Tr), along with an increase in intercellular CO_2_ concentration (Ci). These changes indicate both stomatal and non-stomatal limitations to carbon assimilation, consistent with previous studies (Guo et al., 2018; Shen et al., 2024). Application of H_2_S markedly restored these parameters, suggesting its role in enhancing photosynthetic efficiency under stress (**Figure 9**). This recovery may be attributed to the preservation of chlorophyll content and the stabilization of chloroplast ultrastructure. Salt stress is known to inhibit chlorophyll biosynthesis, leading to reduced Chla, Chlb, and Car contents (Kamran et al., 2020), whereas H_2_S treatment prevented such declines, similar to earlier reports that H_2_S protects chlorophyll from degradation under stress (Tang et al., 2020) (**Figure 7**). Furthermore, the increase in chlorophyll precursors such as Proto IX and Pchl after H_2_S application aligns with evidence that H_2_S promotes chlorophyll synthesis (Wang et al., 2021). Importantly, chlorophyll fluorescence parameters, including Fm and Fv/Fm, which indicate PSII photochemical efficiency, were improved by H_2_S (**Figure 8**). These results suggest that H_2_S protects PSII from salt-induced damage, corroborated by the observed chloroplast ultrastructure integrity. Moreover, the reduction in signal fluorescence intensity under salt stress, implying impaired redox homeostasis and potential damage to the oxygen-evolving complex (Huang et al., 2019), was alleviated by H_2_S, which facilitated electron transfer from PSII reaction centers to acceptors (QA, QB, PQ). A critical consequence of salt stress is the excessive accumulation of reactive oxygen species (ROS), including H_2_O_2_ and O_2_-, which trigger oxidative damage as indicated by enhanced malondialdehyde (MDA) levels and positive DAB/NBT staining. This observation is consistent with earlier reports of ROS-induced cellular injury in plants under salinity (Ozfidan-Konakci et al., 2020; Meng et al., 2024). In our study, H_2_S significantly reduced ROS and lipid peroxidation markers, thereby mitigating oxidative stress in sorghum seedlings (**Figure 2**). This reduction suggests that H_2_S not only restricts ROS overproduction but also enhances the detoxification mechanisms required to maintain cellular redox balance.

The observed alleviation of oxidative stress by H_2_S is closely linked to its regulatory effect on antioxidant metabolism. Under salt stress, the contents of ascorbate (AsA) and glutathione (GSH), as well as their oxidized counterparts (DHA and GSSG), declined, indicating disruption of the ascorbate–glutathione (AsA–GSH) cycle (Chauhan et al., 2021; Bashir et al., 2022; Tan et al., 2022) (**Figure 3**). Additionally, the activities of key enzymes, including APX, GR, DHAR, and MDHAR, were suppressed, further weakening the redox buffering capacity (**Figure 4**). Previous studies have shown that these enzymes are regulated by stress signals and are essential for detoxifying ROS (Pallavi et al., 2012; Jahan et al., 2019), while overexpression of APX in transgenic plants enhances oxidative stress tolerance (Gill and Tuteja, 2010). In our study, exogenous H_2_S restored both the metabolite pools and enzymatic activities, thereby reactivating the AsA–GSH cycle. Furthermore, the cross-talk between the AsA–GSH cycle and other antioxidant systems such as thioredoxin and glutaredoxin may contribute to the overall redox homeostasis (Wu et al., 2019; Luo et al., 2023). Collectively, these findings demonstrate that H_2_S enhances sorghum tolerance to salinity by integrating growth regulation, photosynthetic protection, ROS scavenging, and antioxidant metabolism.

## 5 Conclusion

**Figure 11:**
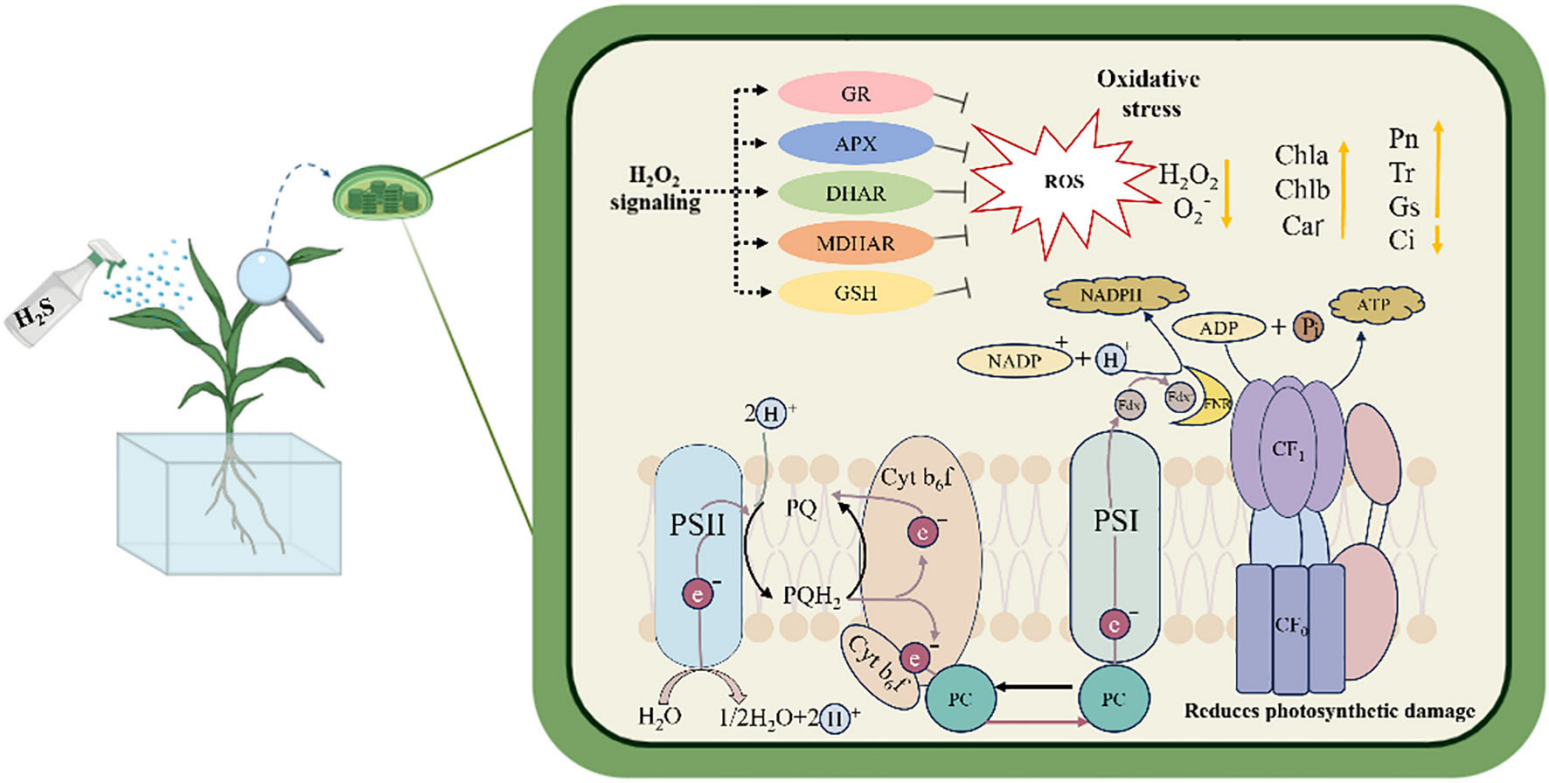
The mechanistic model of H_2_S alleviates salt stress in sorghum seedlings.

This study demonstrated that under salt stress, exogenous application of H_2_S effectively alleviated oxidative stress in sorghum seedlings by activating the AsA-GSH cycle within chloroplasts, thereby maintaining cellular redox homeostasis. Meanwhile, H_2_S preserved chlorophyll content and chloroplast ultrastructure, improved chlorophyll fluorescence parameters, protected photosystem II (PSII) from damage, and facilitated electron transfer from the PSII reaction center to plastoquinone. Collectively, these effects enhanced photosynthetic performance, ultimately mitigating the adverse impacts of salt stress on sorghum seedlings (**Figure 11**).

## Data Availability

The original contributions presented in the study are included in the article/supplementary material. Further inquiries can be directed to the corresponding author.
